# The translation initiation factor eIF2 is phosphorylated to inhibit protein translation through reactive oxygen species under nutrient deficiencies in Arabidopsis

**DOI:** 10.1007/s44154-025-00211-2

**Published:** 2025-01-23

**Authors:** Xiaona Cui, Yuanyuan Cao, Mengyang Lv, Shuhao Zhou, Meijun Chen, Chengwei Li, Hairong Zhang

**Affiliations:** Department of Biochemistry and Molecular Biology, College of Life Sciences, Henan Agricultural University, Zhengzhou, 450046 China

**Keywords:** Acronutrient deprivation, Reactive oxygen species (ROS), General control non-depressible 1 (GCN1), GCN2, eIF2α phosphorylation, Protein translation

## Abstract

Nitrogen (N), phosphorus (P) or potassium (K) deficiency in plants can lead to a decrease in amino acid and protein synthesis. However, it is unknown how protein translation gets repressed during macronutrient deficiencies. Previous research has shown that general control non-depressible 1 (GCN1) cooperate with GCN2 to phosphorylate the alpha subunit of eukaryotic translation initiation factor (eIF2α). In this study, we observed phosphorylation of eIF2α under N, P, and K deficiencies, which was found to be lost in *gcn1*. Mutant *gcn1* displayed higher sensitivity to macronutrient deficiencies compared to the wild-type (WT). The evidence of in situ reactive oxygen species (ROS) accumulation in leaves indicated that macronutrient starvation triggers ROS production. Treatment with Dimethylthiourea (DMTU), a ROS scavenger, eliminated ROS and reversed eIF2α phosphorylation induced by nutrient deficiency. Moreover, it was discovered that protein translation was reduced under N or K deficiency in the WT but not in *gcn1*, whereas under P deprivation, protein translation was reduced in both the WT and *gcn1*. We additionally found that DMTU can partially recover translation inhibition under N or K deprivation. Taken together, it is concluded that GCN1-GCN2-eIF2α pathway is regulated by ROS and is essential for plant survival under macronutrient starvation conditions.

**Supplementary Information:**

The online version contains supplementary material available at 10.1007/s44154-025-00211-2.

## Introduction

When plants encounter nutrient deprivation, they can acclimate nutrient deficiencies by reducing the rate of protein synthesis. Protein turnover has been shown to decrease in *Chlamydomonas reinhardtii* single-cell after transferring it to nitrogen (N) free medium as its net protein synthesis falls to zero after 2 h (Jones et al. 1968). Phosphorus (P) is an important nutrient, as 40–60% of P in plants is allocated to nucleic acids, the majority of which function as ribosomal RNAs (rRNAs) (Veneklaas et al. 2012). Under P deficiency in Arabidopsis, the number of ribosomes is reduced, which in turn decreases protein biosynthesis in mature leaves (Sulpice et al. 2014). During potassium (K) starvation, most amino acids are found to be decreased in K-sensitive genotype of wheat BN207 but increased in K-tolerant genotype of KN9204, indicating that amino acid metabolism is important for plant’s acclimation to K starvation. These research shows that N or P deficiency inhibits protein synthesis and K deficiency can affect amino acid metabolism. However, from now on, it is unknown whether K deficiency can inhibit protein translation and how N, P, or K deficiency inhibits protein synthesis.

In mammals, there are four different kinases of eIF2α (the α subunit of eukaryotic translation initiation factor 2), including general control non-depressible 2 (GCN2), the double stranded-RNA-dependent protein kinase R, the PKR-like endoplasmic reticulum kinase and the heme-regulated eIF2α inhibitor (Baird and Wek 2012; Wek et al. 2006). Translation initiation factor eIF2 contains α, β and γ subunits. In Arabidopsis, eIF2α has two homologies, with over 80% homology between them. The alpha subunits at S51 is the conserved phosphorylation sites in mammals, yeast and plants. Upon different stress stimuli, a corresponding kinase is activated to phosphorylate eIF2α. Phosphorylated eIF2α binds tightly with guanine nucleotide exchange factor eIF2B to inhibit the recycling eIF2-GDP to eIF2-GTP, which restricts the delivery of Met-tRNA_i_^Met^ to ribosomes, thereby significantly reduces global translation initiation.

In yeast and Arabidopsis, only a GCN2 kinase is found (Dever et al. 1992; Lageix et al. 2008). In yeast, GCN1 is essential for the activation of GCN2 (Marton et al. 1993). In *Arabidopsis thaliana*, AtGCN1 and AtGCN2 are respectively homologous with yeast GCN1 and GCN2 (Wang et al. 2017; Zhang et al. 2003). AtGCN1 interacts with AtGCN2 (Wang et al. 2017), and AtGCN2 is activated to phosphorylate eIF2α in response to wound (Lageix et al. 2008), cold (Wang et al. 2017), UV-radiation and amino acid starvation (Marbach et al. 2001; Zhang et al. 2003).

ROS (reactive oxygen species) are messengers to participate in signal transduction (Hasanuzzaman et al. 2020a, 2020b; Sachdev et al. 2021). Naturally, there is a balance between the generation and the scavenging of ROS, and ROS are normally at a lower level to regulate the growth and metabolism (Mittler 2017; Xie et al. 2019). Upon the deprivation of N, P or K nutrient, ROS concentrations increase in Arabidopsis, mainly in the specific region of roots (Shin et al. 2005).

Here, we demonstrated that in Arabidopsis, eIF2α was phosphorylated under N, K and P deficiencies. Due to GCN1 (namely AtGCN1) defect, eIF2α was not phosphorylated in *gcn1* under both normal and nutrient deficiency conditions. Assays were further performed to demonstrate that translation inhibition under N or K deprivation requires eIF2α phosphorylation mediated by GCN1 and the phosphorylation of eIF2α is triggered by the accumulation of ROS.

## Results

### Mutant *gcn1* is hypersensitive to nutrient starvation

**Fig. 1:**
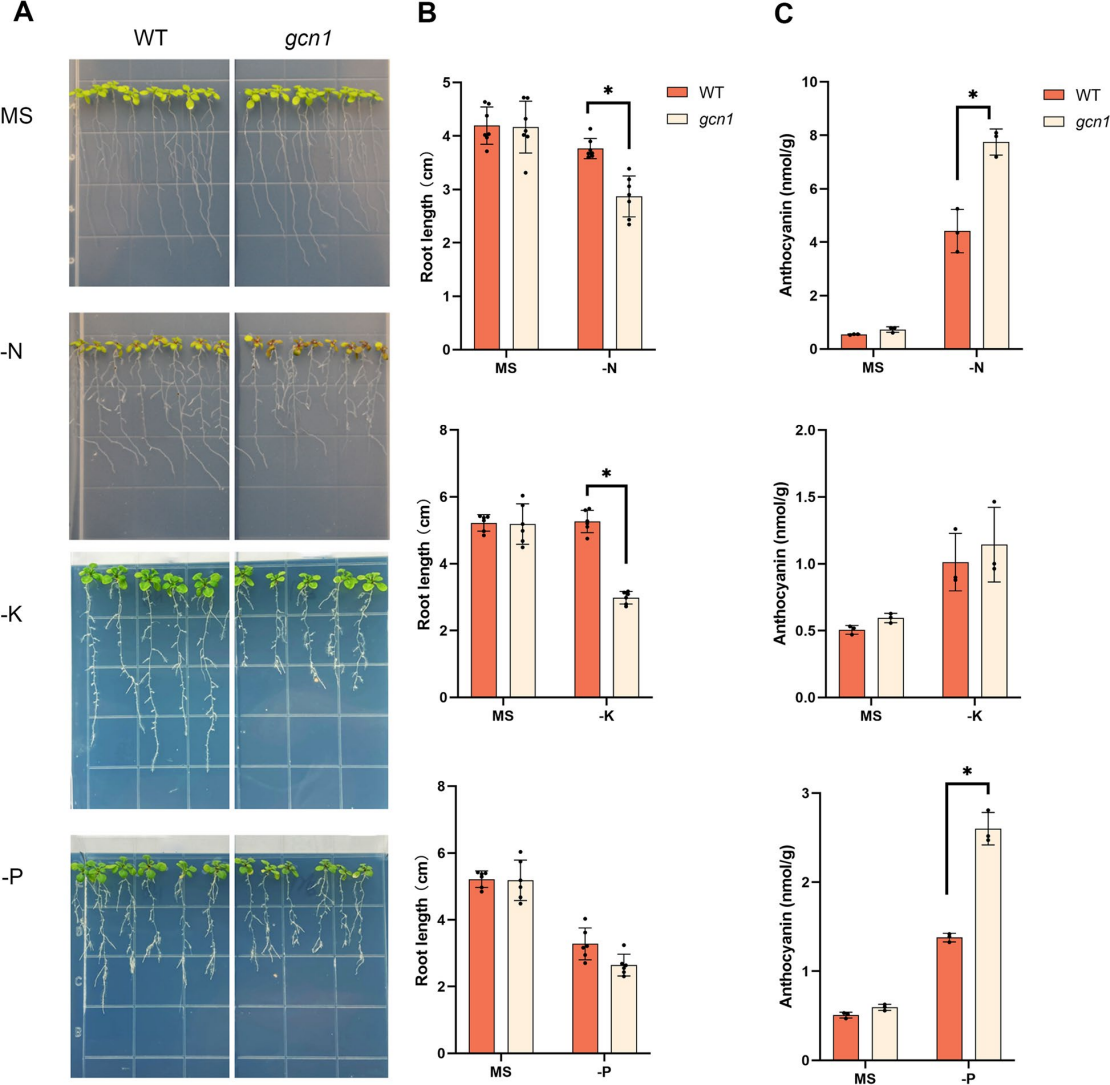
Phenotype of *gcn1* under macronutrient deprivation. **A** Phenotype of *gcn1* under nutrient deprivation. Four-day-old seedlings were transferred to ½ MS plates without different nutrients, and photographs were taken after transferring for 5–7 days. **B** Primary root length under N, K or P deprivation. **C** Anthocyanin content under N, K or P deprivation. Asterisk indicates significant difference between *gcn1* and WT under nutrient deprivation. Data represent mean ± SD (n = 6) for root length, and mean ± SD (n = 3) for anthocyanin content, * P < 0.05, determined using Student’s t-test, compared to WT. MS, ½ MS; -N, ½ MS without N; -K, ½ MS without K; -P, ½ MS without P

It has been found that *gcn1* is more sensitive to cold stress than the WT because of the defects with eIF2α phosphorylation and the slight reduction of mRNA loading onto ribosomes (Wang et al. 2017). To test the function of GCN1 in nutrient deficiencies, we studied the phenotype of *gcn1* under N, P or K deprivation. Four-day-old seedlings were transferred on MS medium without N, P or K. After the transferring for 5 days, the apex and leaves of *gcn1* mutant grown in N-free medium became darker and it was detected that *gcn1* accumulated more anthocyanin than the WT (Fig. 1A and C). Moreover, the root length of *gcn1* was shorter than that of the WT under N starvation conditions (Fig. 1B). In K-free medium, even if the root growth of WT was also hindered, the root length of *gcn1* was much shorter than that of the WT. On the other hand, the anthocyanin content was found to have no difference between the WT and *gcn1* (Fig. 1A, B and C). In P-free medium, the growth of both WT and *gcn1* was severely inhibited (Fig. 1A). However, it was observed that *gcn1* accumulated higher levels of anthocyanin in comparison to WT (Fig. 1A and C). All these results indicated that mutant *gcn1* is hypersensitive to nutrient (N, P or K) starvation.

### GCN1 is essential to phosphorylate eIF2α under nutrient deficiencies

**Fig. 2:**
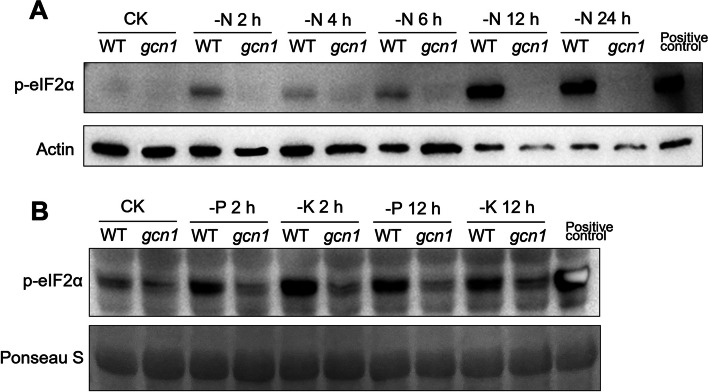
eIF2α was phosphorylated under macronutrient deficiencies. **A** eIF2α was phosphorylated under N starvation. **B** eIF2α was phosphorylated under K and P starvation. Ten-day-old seedlings were transferred to ½ MS liquid medium or medium without different nutrients for indicated times. The phosphorylation level of eIF2α (P-eIF2α) following chlorsulfuron treatments was used as the positive control. -N, N deprivation; -K, K deprivation; -P, P deprivation. The results of immunoblotting assays using the antibody against Actin or ponseau S staining were used as loading controls

The hypersensitivity of *gcn1* to N, P or K starvation indicates that GCN1 is essential for plant’s response to nutrient deficiencies. It has been confirmed that GCN1 interacts with GCN2 and is required for activating GCN2 to phosphorylate eIF2α in cold stress (Wang et al. 2017). We detected the phosphorylation level of eIF2α under macronutrient starvation. Under N starvation, it was observed that eIF2α phosphorylation was induced at 2, 4, 6, 12 and 24 h treatment, although eIF2α phosphorylation level was slightly declined at 4 and 6 h (Fig. 2A). For K or P deprivation treatments, a high level of eIF2α phosphorylation was noticed in both 2 h and 12 h of nutrient starvation (Fig. 2B). In *gcn1* mutant, the activity of GCN2 keeps a low level, so the level of eIF2α phosphorylation was at background levels in all nutrient deprivation treatments (Fig. 2). Seedlings sprayed with chlorsulfuron (CHR) solution were used for protein extraction and set as positive control of p-eIF2α. These results showed that macronutrient starvations can induce eIF2α phosphorylation and GCN1 is essential for GCN2 activation to phosphorylate eIF2α under macronutrient deprivation.

### Nutrient deprivation induces ROS accumulation in leaves

**Fig. 3:**
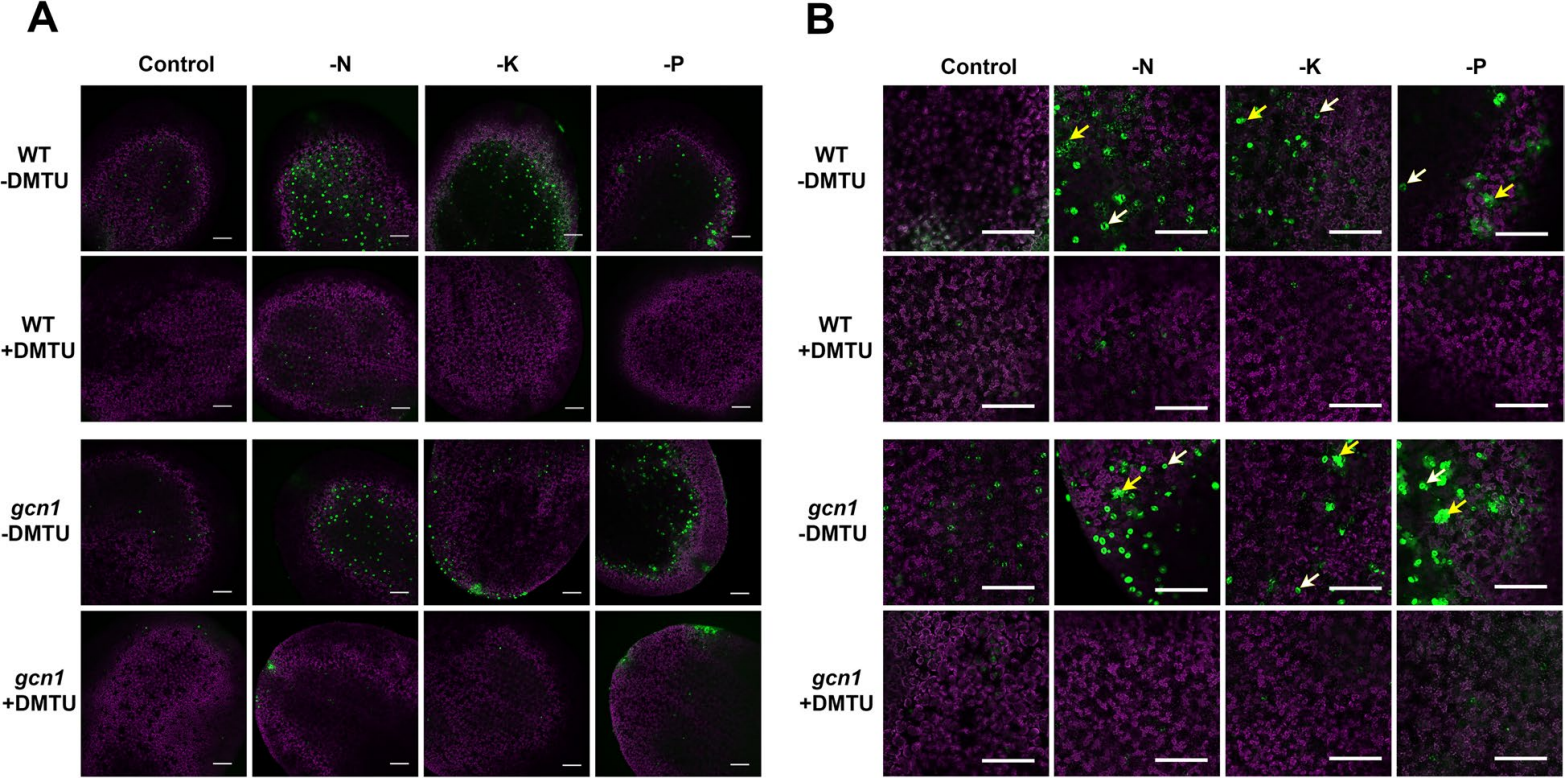
ROS accumulated under macronutrient deprivation. **A** Ten-day-old seedlings were transferred into ½ MS liquid medium or medium without different nutrients for 10 h. ROS were detected using the H_2_DCFDA fluorescence probe. Fluorescence signals in the cotyledon cells were examined using laser confocal microscopy. **B** Enlarged views of cotyledons revealed the ROS signals in guard cells (indicated by the white arrow) and the leaf mesophyll cells (indicated by the yellow arrow). The notations -N, -K, and -P refer to nitrogen, potassium, and phosphorus deprivation, respectively. Scale bars indicate 200 μm

Macronutrient starvation induces GCN1-GCN2 activation to phosphorylate eIF2α, but how GCN1-GCN2 is being activated remains unclear. Recently, it is reported that the chloroplast can assimilate light to produce ROS and ROS can further activate GCN2 to phosphorylate eIF2α (Lokdarshi et al. 2020). It is noticed that ROS can accumulate in roots in response to N, K or P deprivation (Shin et al. 2005). We proposed that macronutrient starvation induces ROS accumulation in cytoplasm and accumulated ROS in turn activate GCN1-GCN2 to phosphorylate eIF2α. The fluorescent probe 2’,7’-dichlorofluorescein diacetate (H_2_DCFDA) was used to detect various forms of ROS, primarily H_2_O_2_ but also including hydroxylradicals and superoxide anions (Fichman et al. 2019; Val-Torregrosa et al. 2022). We investigated ROS signals under nutrient deprivation using the H_2_DCFDA probe. Ten-day-old seedlings were respectively transferred to medium without N, K or P. After 10 h of nutrient deprivation, the seedlings were incubated by H_2_DCFDA solution for ROS detection. Fluorescence in cotyledon cells was detected using laser confocal microscope (Fig. 3). The green fluorescence corresponds to ROS signal, while the purple fluorescence represents chlorophyll autofluorescence (Fig. 3). Under all macronutrient starvation conditions, there was a significant increase in ROS signals in both WT and *gcn1* mutants seedlings compared to those grown under normal conditions (Fig. 3A). Notably, the ROS signals were predominantly localized in the guard cells; however, under macronutrient starvation, there were also observable accumulation of ROS in the leaf mesophyll cell (Fig. 3B). Additionally, the intensity of the ROS signals was diminished following treatment with dimethylthiourea (DMTU), a known ROS scavenger. These findings suggest that deprivation of macronutrients leads to the accumulation of ROS in leaves, as well as in roots (Shin et al. 2005).

### Phosphorylation of eIF2α under nutrient deprivation depends on ROS accumulation

**Fig. 4:**
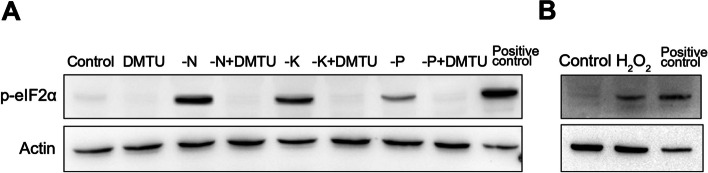
The phosphorylation of eIF2α under macronutrient deprivation is dependent on ROS. **A** The phosphorylation of eIF2α induced by macronutrient deprivation was lost after treatments with a ROS scavenger DMTU. Ten-day-old seedlings were transferred to ½ MS liquid medium or medium without different nutrients for 2 h. -N, N deprivation; -K, K deprivation; -P, P deprivation; + DMTU, 10 mM DMTU was added into ½ MS medium. **B** The phosphorylation of eIF2α under H_2_O_2_ treatments. H_2_O_2_, the solution of 100 mM H_2_O_2_ was sprayed onto the leaves of seedlings for 1 h. The western blot results using the antibody against Actin were used as loading controls

Upon macronutrient deprivation, eIF2α was phosphorylated and ROS were accumulated (Figs. 2 and 3). To test the correlation between eIF2α phosphorylation and ROS accumulation under nutrient deprivation, we placed ten-day-old seedlings in macronutrient free medium supplied with or without DMTU, a well-established scavenger to eliminate cytoplasmic H_2_O_2_ (Curtis et al. 1988). After 2 h of treatments, seedlings were collected for ROS staining and protein extraction. In macronutrient free medium supplied with DMTU, DMTU eliminated most of the ROS as expected (Fig. 3). Extracted proteins were used to analyze the level of eIF2α phosphorylation. Western blot results showed that the level of eIF2α phosphorylation increased under macronutrient deprivation, but fell to background levels under DMTU treatments (Fig. 4A). It was confirmed that H_2_O_2_ treatments can lead to the phosphorylation of eIF2α (Fig. 4B), which is consistent with a previous report (Lokdarshi et al. 2020). The correlation between eIF2α phosphorylation and ROS signals suggested that nutrient deprivation stimulates ROS accumulation and accumulated ROS in turn activate GCN1-GCN2 to phosphorylate eIF2α.

### De novo protein synthesis correlates with the level of eIF2α phosphorylation under nutrient deficiencies

In Arabidopsis, GCN2 phosphorylates eIF2α to globally inhibit ribosome loading onto mRNA under amino acid deprivation (Lageix et al. 2008; Lokdarshi et al. 2020). Under cold stress conditions, it is also noticed that GCN1-mediated GCN2 activation slightly decreases ribosome loading onto mRNA (Wang et al. 2017). The defect of eIF2α phosphorylation along with *gcn1’s* hypersensitivity to nutrient deprivation suggested that GCN1-mediated eIF2α phosphorylation represses protein translation to meet nutritional deficiency during starvation.

Ribosome profiling are usually used to study mRNA translation states (Juntawong et al. 2014; Lageix et al. 2008; Lokdarshi et al. 2020; Nicolai et al. 2006; Sulpice et al. 2014). Ribosome profiling under N, K and P starvation were obtained from the OD_260_, which measuring mRNA content when ribosomes were fractionated by sucrose density gradient. The ratios of polysome/monosome showed that CHR inhibited ribosome loading to mRNA in the WT, but not in *gcn1* (Fig. S1A and S1B), which is consistent with the report in *gcn2* as described (Lokdarshi et al. 2020). Under N deprivation conditions, mRNA translation levels were increased after 2 h and only slightly decreased after 12 h (Fig. S1A, S1C and S1D). Similar results were also acquired under K and P starvation conditions, as the ratios of polysome/monosome are not decreased after 2 h K or P deprivation, and even increased after 12 h (Fig. S2). Moreover, although eIF2α phosphorylation is impaired, mRNA translation states in *gcn1* are lower than that in the WT under macronutrient deficiencies conditions. The results suggest that mRNA translation states in plants are affected by not only eIF2α phosphorylation but other ways, such as translation elongation and ribosome formation, and ribosome profiling can’t be used to indicate translation levels under N, K and P starvation conditions.

**Fig. 5:**
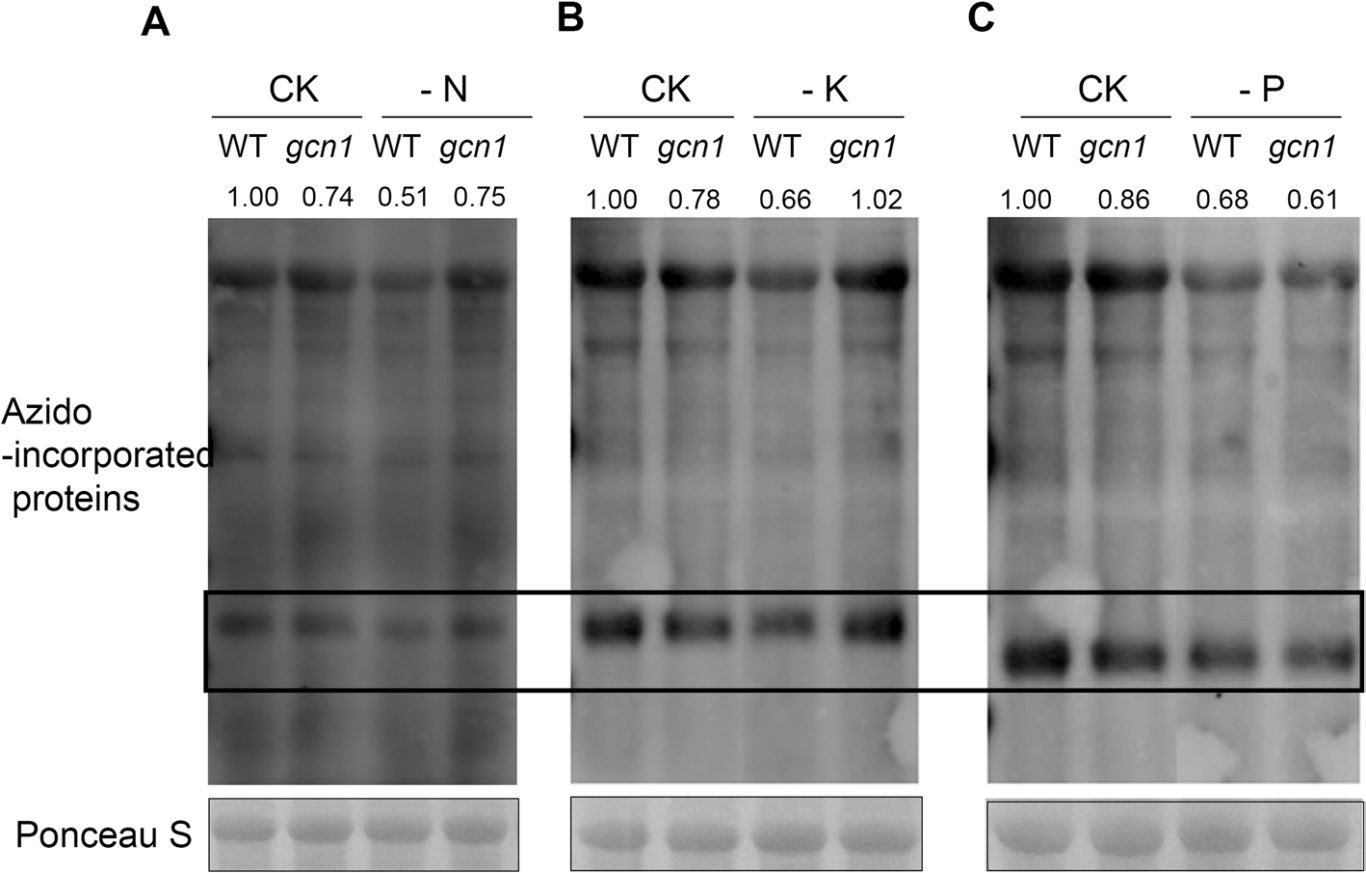
De novo protein synthesis of *gcn1* mutants under macronutrient deficiencies. Ten-day-old seedlings were transferred to liquid ½ MS medium or medium without N (**A**), K (**B**) or P (**C**) for 2 h. 50 μM AHA was contained in the medium. -N, N deprivation; -K, K deprivation; -P, P deprivation. Ponseau S staining indicates protein loading amounts. In the box are the bands of Rubisco small subunits

We then checked newly synthesized proteins in *gcn1* under nutrient deprivation. L-azidohomoalanine (AHA) is an analogue of methionine, containing an azide group. When Arabidopsis seedlings were transferred onto MS medium containing AHA, AHA was incorporated into nascent proteins. The azide moiety of AHA can be ligated with an alkyne. Azido-incorporated proteins were thus detected with alkyne fluorescent by using western blot. The Rubisco small subunit (RbcS) bands of western blot results were calculated in Image J software. The western blot results showed that protein synthesis was obviously inhibited under N, P, and K deprivation conditions in the WT (Fig. 5A, B and C). However, there was no significant reduction in the amount of newly synthesized protein under N or K starvation conditions in *gcn1* mutant. Protein translation in *gcn1* is not inhibited under N or K starvation, which indicates that translation inhibition under N or K starvation depends on GCN1-mediated eIF2α phosphorylation. On the other hand, under P deprivation, protein translation was inhibited in both the WT and *gcn1* mutant and there was no difference in the level of nascent proteins between the WT and *gcn1*. The non-difference demonstrated that protein translation under P starvation can be inhibited by other unknown means, independent of eIF2α phosphorylation.

**Fig. 6:**
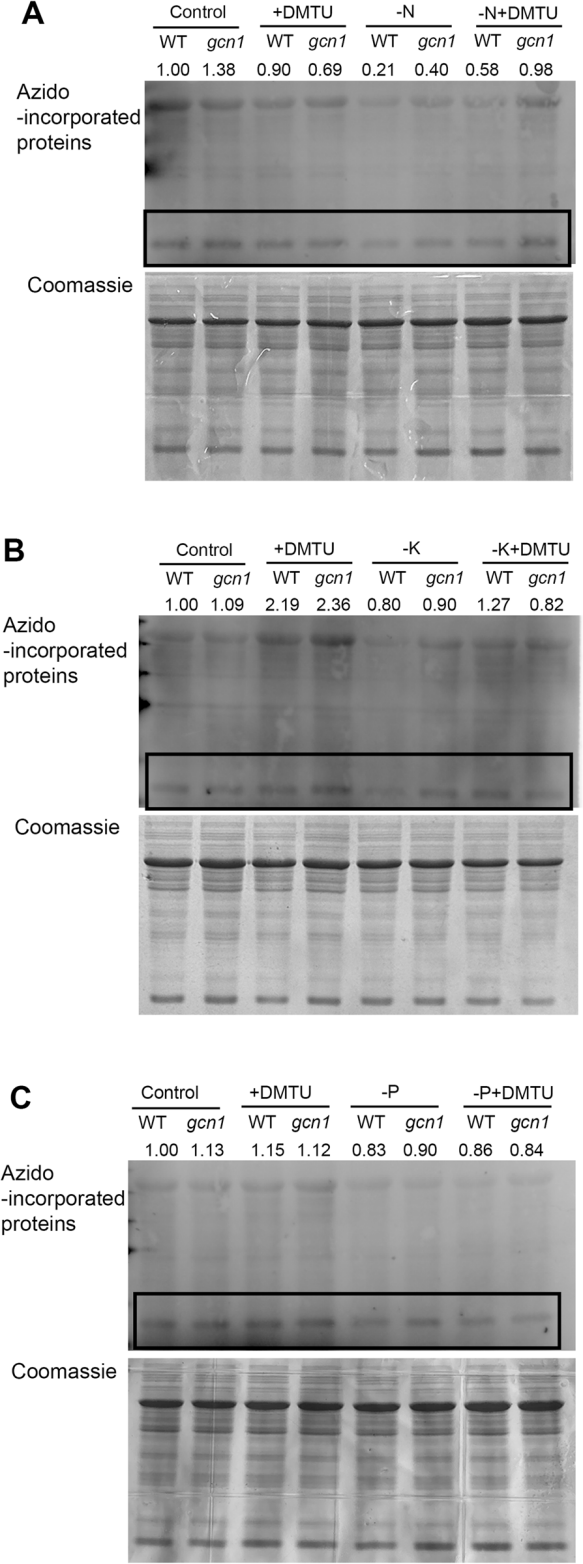
Recovery of de novo protein synthesis by DMTU treatments under macronutrient deficiencies. Ten-day-old seedlings were transferred to liquid ½ MS medium or medium without N (**A**), K (**B**) or P (**C**) for 2 h. 50 μM AHA was contained in the medium. -N, N deprivation; -K, K deprivation; -P, P deprivation; + DMTU, 10 mM DMTU was added into MS medium. Coomassie staining indicates protein loading amounts. In the boxes are the bands of Rubisco small subunits

As a ROS scavenger, DMTU inhibits eIF2α phosphorylation under nutrient deprivation (Fig. 4). Newly synthesized proteins detected by azido-modified proteins demonstrated that the translation inhibition by eIF2α phosphorylation under N or K deprivation was partially recovered by DMTU treatments (Fig. 6A and B). The recovery of nascent proteins by DMTU treatments confirmed that eIF2α phosphorylation mediated by ROS is the main way to inhibit protein translation under N or K starvation. On the other hand, the inhibition under P deprivation was not depressed by DMTU treatments (Fig. 6C). The non-recovery confirmed that there are other pathways, besides eIF2α phosphorylation, to reduce protein translation under P deprivation.

## Discussion

**Fig. 7:**
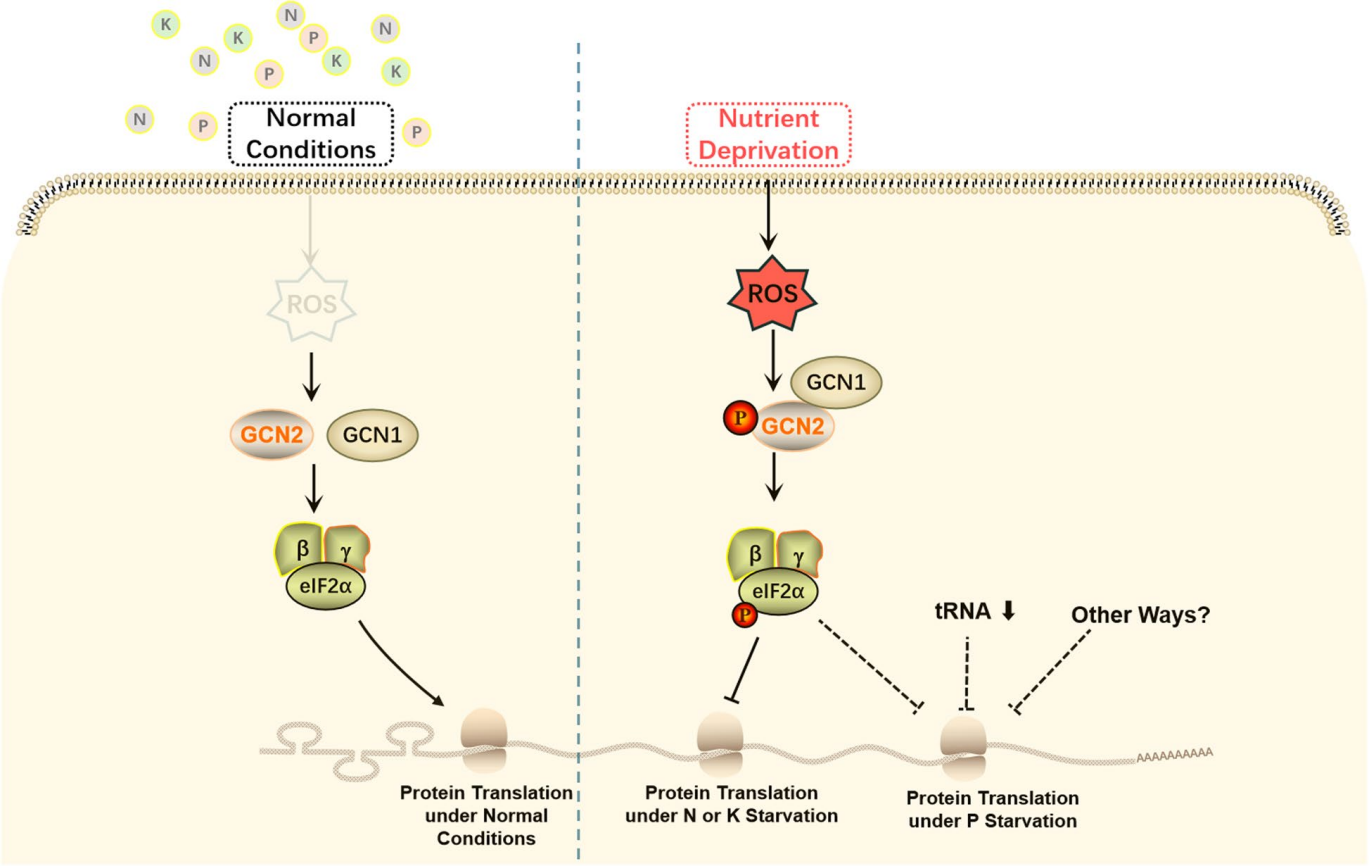
The regulation of protein translation in normal or macronutrient starvation conditions. Under normal conditions, the amount of ROS keeps at a low level, GCN2 is inactivated, no eIF2α is in phosphorylated form and proteins are quickly translated to meet growth demand. When plants encounter macronutrient starvation, ROS is highly accumulated, GCN2 is activated through interacting with GCN1 by ROS, the amount of phosphorylated eIF2α is greatly increased. Under N or K starvation, protein translation is blocked through eIF2α phosphorylation for adaption to the stress condition of nutrient starvation. Under P starvation, there should be other ways, such as the reduction of rRNA formation, to inhibit protein translation, in addition to eIF2α phosphorylation

In yeast, GCN2 is a sensor of amino acid deficiency in starvation conditions, in which uncharged-tRNAs bind to a HisRS domain of GCN2 and activate it (Deval et al. 2009; Donnelly et al. 2013). In our previous research, we have demonstrated that the C-terminal of GCN1 combines with GCN2, which is essential for GCN2 activation to phosphorylate eIF2α in Arabidopsis (Wang et al. 2017). The results in this research demonstrated that accumulated ROS under macronutrient deprivation activate GCN1 and GCN2 complex to phosphorylate eIF2α, which further inhibits protein translation under N or K deficiencies (Fig. 7). To our knowledge, it is the first report on how translation is suppressed under macronutrient deprivation.

In plants ROS play a significant role in stress response by transferring signaling (Hasanuzzaman et al. 2020b; Mittler 2017; Sachdev et al. 2021). It was observed that an enormous amount of ROS was accumulated in leaves under nutrient starvation (Fig. 3). Under the conditions of N, K and P starvation, the phosphorylation of eIF2α was observably increased (Fig. 2). After ROS were scavenged by DMTU, no eIF2α in phosphorylated form was detected (Fig. 4). The relationship between the amount of ROS and eIF2α phosphorylation suggested that ROS accumulation is essential for activating GCN1-GCN2 to phosphorylate eIF2α under nutrient starvation. Nascent proteins were reduced under nutrient deprivation but DMTU treatments could partially recover translation inhibition under N or K deprivation (Figs. 5 and 6), indicating that eIF2α phosphorylation induced by ROS accumulation plays a key role to inhibit protein translation during N or K starvation. The research discovered firstly that ROS accumulation is required for eIF2α phosphorylation in N or K deprivation.

Under amino acid starvation condition, eIF2α phosphorylation reduces ribosome loading onto mRNA to save energy in both yeast and *Arabidopsis thaliana* (Dong et al. 2000; Masson 2019). It is well known that protein translation consumes a lot of energy, so it is reasonable to find that there were less newly synthesized proteins in nutritional deprivations than in normal conditions (Fig. 5). However, under N or K deprivation, the level of newly synthesized proteins was not decreased in the *gcn1* mutant (Fig. 5A and B). The non-reduction of nascent proteins in *gcn1* and the hypersensitivity of *gcn1* to N or K deprivation (Fig. 1) displays that translation inhibition mediated by eIF2α phosphorylation is essential for plants to survive in N or K deprivation conditions.

On the other hand, the level of newly synthesized proteins in P deprivation conditions was decreased in *gcn1*, as well as in the WT (Fig. 5C). The reduction of nascent proteins in *gcn1* under P deprivation indicates that eIF2α phosphorylation mediated by GCN1 is not the only way to inhibit protein translation during P deprivation conditions. There could be other mechanisms to repress protein translation during P starvation. We suspected that rRNAs could contribute to this repression as P is needed to form nucleic acids into rRNAs (Veneklaas et al. 2012), which is also supported by the proteome data after P starvation (Mehta et al. 2021). Although eIF2α phosphorylation is not the only pathway to inhibit protein translation under P deprivation, ROS accumulation and eIF2α phosphorylation in P deprivation along with the hypersensitivity of *gcn1* to P deprivation indicated that eIF2α phosphorylation mediated by GCN1 is important for plant’s adaptability to P deprivation. In addition to the general inhibitory effect on translation, eIF2α phosphorylation may selectively regulate the transcription or translation of specific genes (Cui et al. 2021), which may explain why *gcn1* is more sensitive to P deprivation than the WT. However, which genes are selectively regulated by GCN1 in P deprivation need be further researched.

In the AHA-labeled nascent proteins, the amounts of Rubisco small subunit were calculated to indicate the translation level (Figs. 5 and 6). Rubisco large subunit (RbcL) and RbcS combine together to be a mature Rubisco, a rate limiting enzyme of photosynthesis in chloroplast matrix (Vitlin Gruber and Feiz 2018). It is plausible that the cytosolic RbcS was regulated by GCN1-GCN2 mediated eIF2α phosphorylation under nutrient deprivation. However, as it was observed, the plastid-encoded protein RbcL, which was synchronously regulated as RbcS. The lessening of plastid-encoded RbcL indicates that RbcL and RbcS may be regulated in chain by unknown way, as these two proteins always work together.

In yeast and mammals, a small fraction of the eIF2α phosphorylation can hinder eIF2B from converting eIF2α-GDP into eIF2α-GTP, thereby interrupting translation initiation under stress conditions (Baird and Wek 2012; Rowlands et al. 1988; Scorsone et al. 1987). In Arabidopsis, eIF2α phosphorylation by GCN2 inhibit ribosome loading to mRNA under amino acid deprivation (Lageix et al. 2008; Lokdarshi et al. 2020). However, under cold stress or H_2_O_2_ treatments, although eIF2α phosphorylation is dramatically induced in the WT, ribosome loading to mRNA is inhibited in both the WT and the mutants of *gcn1* or *gcn2*, in which eIF2α phosphorylation is impaired (Lokdarshi et al. 2020; Wang et al. 2017). Under light treatments, ROS emanating from the chloroplast induce eIF2α phosphorylation, but the translation level in the light conditions is higher than that in the dark conditions (Lokdarshi et al. 2020). The non-correlation of translation levels and eIF2α phosphorylation indicates that eIF2α phosphorylation is neither sufficient nor necessary for translation inhibition in plants, at least under cold stress, H_2_O_2_ and high light treatments. The evidences in the research show that eIF2α phosphorylation is essential for translation inhibition under N or K starvation, but not under P deprivation. The importance of eIF2α phosphorylation for translation inhibition varies under different stress conditions in plants, which is different from that in yeast and mammals.

## Conclusion

Collectively, the research demonstrates that macronutrient deficiencies trigger plants to accumulate enormous ROS in leaves and ROS stimulate GCN1 and GCN2 complex to phosphorylate eIF2α. Under N or K starvation, eIF2α phosphorylation is required to inhibit protein translation. Under P deprivation, there should be other ways to repress protein translation, in addition to eIF2α phosphorylation. No matter whether eIF2α phosphorylation is the only way to inhibit protein translation, eIF2α phosphorylation is essential for plants to survive through macronutrient deficiencies.

## Methods and materials

### Materials and growth conditions

The mutant *gl1* (*Arabidopsis thaliana* ecotype Col-0) was used as the background of the WT. Mutant *gcn1* (previously named as *gcn1-1*) was obtained by mutagenizing from *gl1* (Wang et al. 2017). Seeds were spotted on plates with half strength of MS. MS medium: MS powders (PhytoTech Murashige & Skoog Basal Medium with Vitamins, M519), 1% sucrose and 1% agar (Solarbio A8190). After stratification at 4℃ for 48 h, plates were vertically put in climate chamber with 16 h light and 8 h dark cycles at 22℃ and began dark cycle at 23 o’clock of the day.

For nutritional deprivation conditions, 4-day-old seedlings were respectively transferred to ½ MS plates without nitrogen (PhytoTech Murashige & Skoog Modified Basal Salt Mixture with Vitamins and without N, M531-N), potassium (Coolaber Murashige & Skoog Medium with Vitamins and without K, PM1011-K) or phosphorus (Coolaber Murashige & Skoog Medium with Vitamins and without P, PM1011-P), and grown in climate chamber. Photographs of the seedlings after nutritional deprivation treatments for 5–7 days were obtained with digital camera (Nikon D7000).

For nutritional starvation, 10-day-old seedlings were respectively transferred to ½ MS liquid medium without nitrogen, potassium or phosphorus for indicated times. To avoid the hypoxia effect, the roots of seedlings were submerged in the liquid medium while the shoots were in the air, using a home-made loop. At the same time, seedlings as control treatments were transferred to ½ MS liquid medium. All treated seedlings were collected at 17 o’clock, except that for 12 h’s treatments, seedlings were obtained at 21 o’clock before the dark cycle of the day.

For stress treatments with H_2_O_2_ and chlorsulfuron, 10-day-old seedlings in plates were respectively sprayed with 100 mM H_2_O_2_ or 0.5 mM chlorsulfuron and maintained for 1 h. For control treatments, plants were sprayed with 0.1% DMSO, which used to dissolve chlorsulfuron.

### Root length and anthocyanin measurement

The lengths of root were measured in imageJ 1.53 k software. Anthocyanin was detected by using 0.1 mol/L hydrochloric acid ethanol solution (Dilute 8.5 ml of concentrated hydrochloric acid with ethanol to 1000 ml) to extract at 50℃ for 30 min with gentle shaking. The absorbance values at 530 nm, 620 nm and 650 nm were obtained respectively. The formula of was used to calculate the absorbance of ${OD}_{\lambda}=\left({OD}_{530}-OD_{620}\right) -0.1\left(OD_{650}-OD_{620}\right)$ anthocyanin, and then using $\eta (nmol/g)=\frac{{OD}_{\gimel }}{\upepsilon }*\frac{V}{m}*1000000$ (ℇ: 4.62 × 10^6^, Anthocyanin molar extinction coefficient. *V* (mL): Volume of extraction buffer. m (g): fresh weight) to calculate the content of anthocyanin.

### Protein extraction and western blot

Proteins were extracted as previously described with slight modification (Lokdarshi et al. 2020). Briefly, 100 mg 10-day-old seedlings were harvested in 1.5 mL tubes, immediately dipped into liquid nitrogen and ground into fine powder. Tubes with ground powder was added into 120μL protein extraction buffer, containing 25 mM Tris–HCl (pH 7.5), 75 mM NaCl, 5% (v/v) glycerol, 0.05% (v/v) Nonidet P-40, 0.5 mM EDTA, 0.5 mM EGTA, 2 mM DTT, 1 × protease inhibitor cocktail Tablets (Roche cOmplete Tablets) and 1 × protease and phosphatase inhibitor cocktail (Thermo Fisher Scientific; cat. no. PIA32959). Extracted proteins were quantified by Bradford reagent (Bio-Rad Quick start Bradford 1 × Dye).

For western blot analysis, 20 μg of total proteins was separated on a 12.5% SDS-PAGE gel and electroblotted onto a polyvinylidene fluoride (PVDF) membrane for 1 h at 4℃ (GE Amersham Hybond 0.45 μm PVDF, Cat. No. 10600023). Membranes with proteins were blocked in TBST (10 mM Tris Base, pH = 8.0, 150 mM NaCl, 0.05v/v Tween-20) with 1% BSA at 4℃, incubated for 12 h at 4℃ with the antibody (phosphor-eIF2α antibody, Abmart TA3087, diluted to 1:1000 in TBST buffer; alpha-Actin-2 antibody, Abmart, M20009, diluted to 1:10,000), washed 5 times with 1 × TBST each for 5 min, and incubated with anti-rabbit IgG (a 1:10,000 dilution) for 2 h at room temperature. After washing 5 times, signals were obtained with ECL (abkkin Sper Lumia HRP substrate kit, K22020) and captured by CCD imaging system (Tanon 4600).

### ROS detection

After nutrient starvation, 10-day-old seedlings were submerged in PBS buffer (136 mM NaCl, 2.6 mM KCl, 10 mM Na_2_HPO_4_, 2 mM KH_2_PO_4_, pH7.2–7.5) with 20 μM H_2_DCFDA (MCE, cat. no. HY-D0940) for 20 min, and then washed with PBS without H_2_DCFDA. Seedlings were shielded from light exposure until the detection of ROS using laser confocal microscopy (Nikon A1HD25). The ROS signal was detected at a wavelength of 488 nm, while chloroplast autofluorescence was observed at 638 nm. 20 mM DMTU was added into ½ MS medium.

### Newly synthesized protein detection

Newly synthesized proteins were detected according to the manual of Thermo Fisher Click-iT AHA Alexa Fluor 488 Protein Synthesis HCS Assay (Cat. No. c10289). Briefly, 10-day-old seedlings were grown in liquid ½ MS with 50 μM AHA (L-azidohomoalanine, component A) for 2 h during nutrient starvation. AHA-labelled proteins were extracted by protein extraction buffer, electrophoresed in SDS-PAGE and transferred onto PVDF membranes. 300μL 1 × Click-iT reaction cocktail (10 × Click-iT AHA buffer additive by diluting 1:100 in Click-iT supermix) was dropped onto PVDF membranes for 30 min reaction. Images were captured at 488 nm by CCD imaging system (GE AI600). The same amount proteins were loaded, electrophoresed in SDS-PAGE and stained by coomassie blue as loading controls. Alternatively, PVDF membranes with transferred proteins were stained by ponseau S as loading controls. The amounts of the brightest Rubscio proteins in a rectangle were calculated by ImageJ software to represent the amount of newly synthesized proteins.

### Ribosome extraction and fractionation

Ribosomes were extracted, centrifuged and fractionized as reported (Sormani et al. 2011; Wang et al. 2017). Polysome/monosome ratios were calculated as previously described (Lokdarshi et al. 2020). All experiments were repeated for at least three times with similar results.

## Supplementary Information

Supplementary Material 1.

## Data Availability

All data generated or analyzed in this study are presented in this published article and its supplementary data files.

## Abbreviations

AHA: L-azidohomoalanine
CHR: Chlorsulfuron
DMTU: Dimethylthiourea
eIF2α: The alpha subunit of eukaryotic translation initiation factor
GCN1: General control non-depressible 1
GCN2: General control non-depressible 2
K: Potassium
N: Nitrogen
P: Phosphorus
ROS: Reactive oxygen species
WT: Wild-type
