# Supplementary material for: The translation initiation factor eIF2 is phosphorylated to inhibit protein translation through reactive oxygen species under nutrient deficiencies in Arabidopsis
Source: Stress Biol. 2025 Jan 23;5(1):7. doi: 10.1007/s44154-025-00211-2 (PMC11754583; doi:10.1007/s44154-025-00211-2)
Supplement: Supplementary file 1 — Supplementary Material 1. [file 44154_2025_211_MOESM1_ESM.docx]

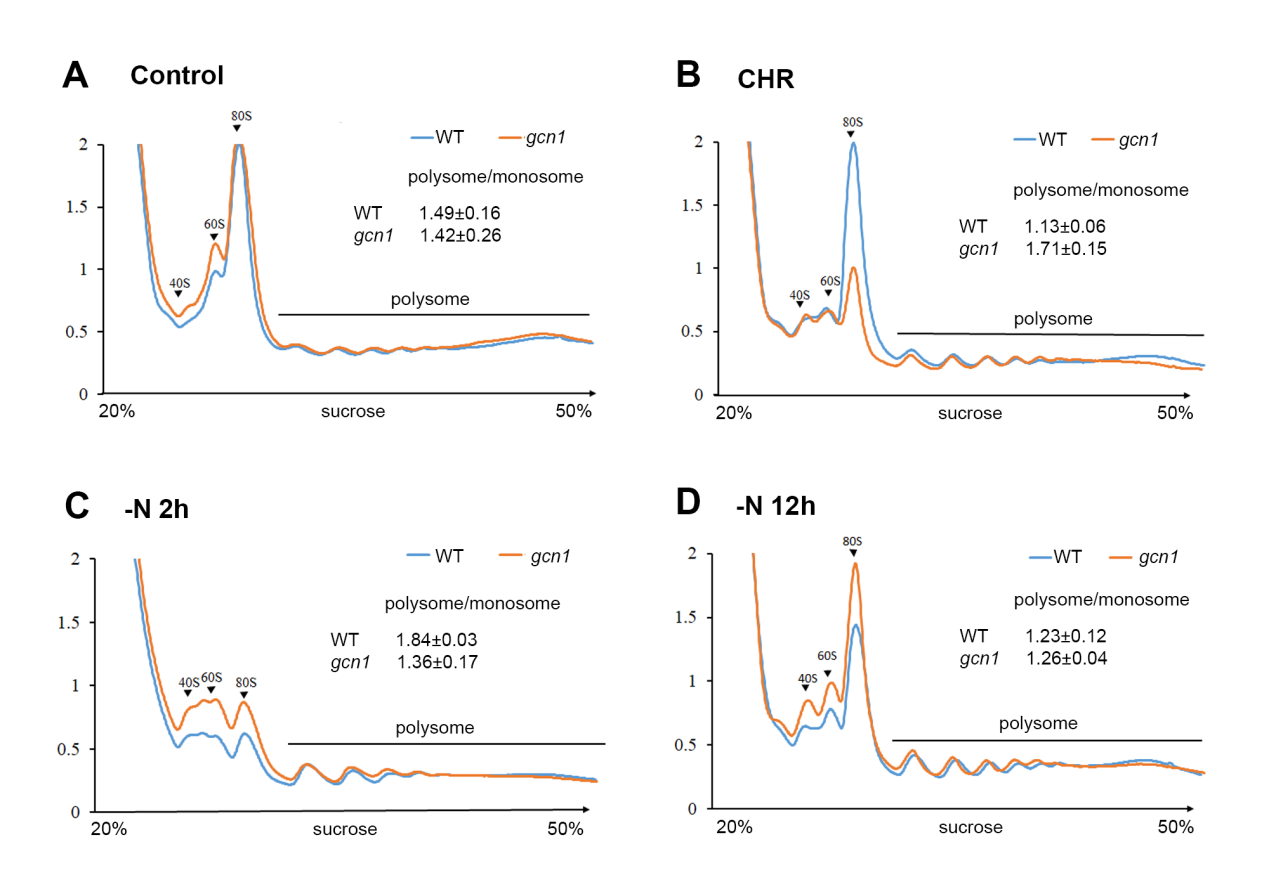


**Fig. S1** The absorbance profiles at 260nm of ribosome gradients after N starvation. Ribosome profiling was respectively captured from plants in normal growth conditions as controls (CK) (**A**), after CHR treatments (**B**), after 2h N starvation (**C**) or after 12h N starvation (**D**). The ratio of polysomes/monosomes is indicated with SE from two replicates. Arrows indicates individually 40S subunits, 60S subunits and 80S ribosomes as shown, and areas below straight lines represent polysomes.


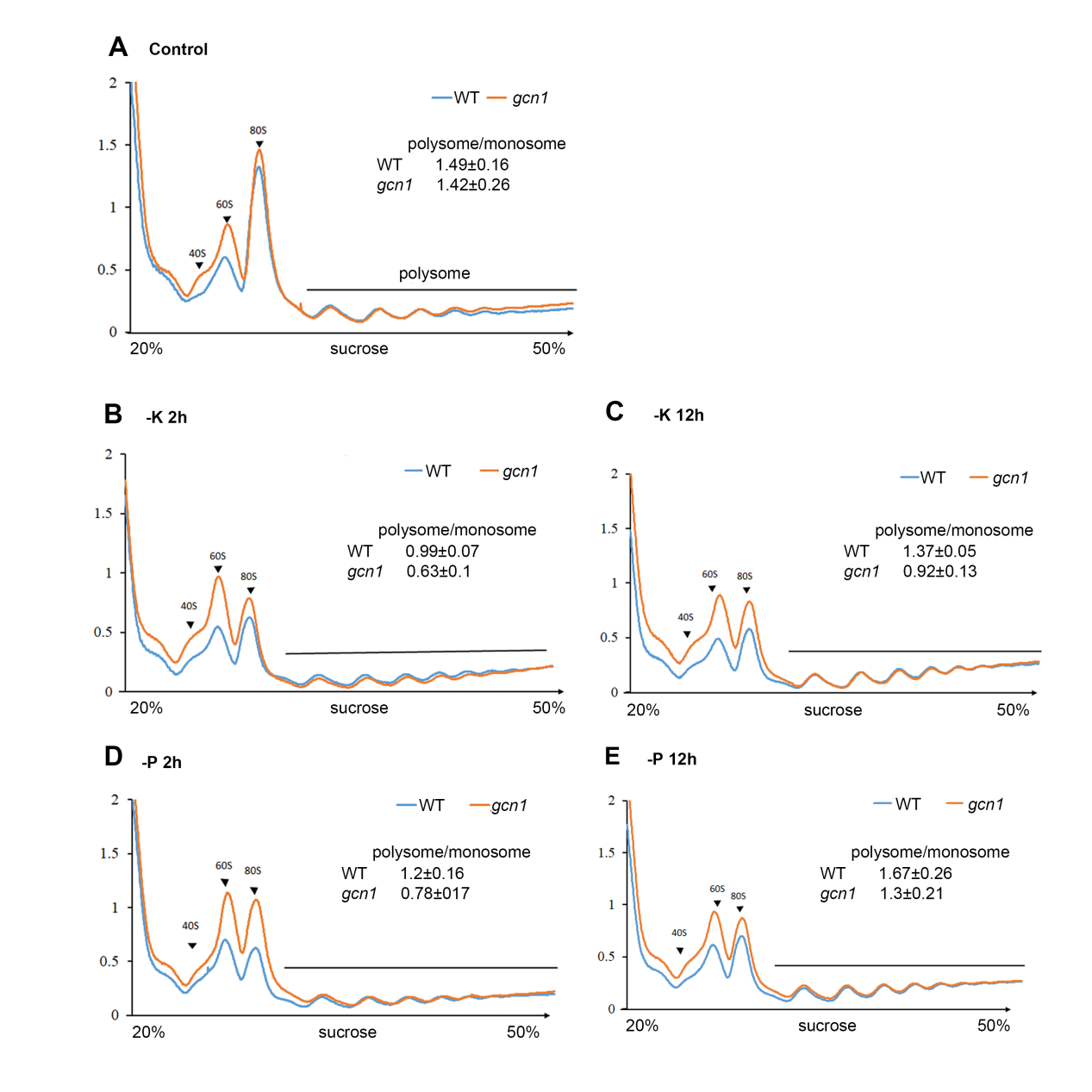


**Fig. S2** The absorbance profiles at 260nm of ribosome gradients after K or P starvation. Ribosome profiling was respectively captured from plants in normal growth conditions as controls (CK) (**A**), after 2h K starvation (**B**), after 12h K starvation (**C**), after 2h P starvation (**D**) or after 12h K starvation (**E**). The ratio of polysomes/monosomes is indicated with SE from two replicates. Arrows indicates individually 40S subunits, 60S subunits and 80S ribosomes as shown, and areas below straight lines represent polysomes.
